# Supplementary material for: The extraperitoneal French AmbUlatory cesarean section technique leads to improved pain scores and a faster maternal autonomy compared with the intraperitoneal Misgav Ladach technique: A prospective randomized controlled trial
Source: PLoS One. 2021 Jan 22;16(1):e0245645. doi: 10.1371/journal.pone.0245645 (PMC7822305; doi:10.1371/journal.pone.0245645)
Supplement: S1 File — (DOCX) [file pone.0245645.s003.docx]

Tunis, le 10.04.2020

Chère professeure Lamia Ben Jemaa , présidente du comité d’éthique hôpital Mongi dlim , La Marsa .

Nous venons par la présente solliciter l’avis et l’accord du comité d’éthique de l hôpital hospitalo universitaire MONGI SLIM LA MARSA concernant la tenue d’une étude clinique interventionnelle randomisée .

Lieu de l’étude : Service de gynécologie obstétrique hôpital Mongi Slim La Marsa

Responsable de l’étude : Pr Ag. Kaouther Dimassi

Période de l’étude estimée 6 mois , Début aout 2018

**PRESENTATION DE L’ETUDE**

**TITRE :**

**ETUDE INTERVENTIONNELLE RANDOMISEE : COMPARAISON ENTRE DEUX TECHNIQUES DE CESARIENNES : FAUCS Vs. MLC**

La césarienne (CS) est l’intervention chirurgicale la plus pratiquée en obstétrique et la plus ancienne en matière de chirurgie abdominale .

Jusqu'au 17^ème^ siècle, la césarienne était une opération exclusivement mortelle pour la mère, indiquée pour sauver la vie du nouveau-né . Jusqu’à la fin du 19^ème^ siècle, l’obstétrique a été dominée par la version par manœuvre interne, par l’utilisation d’instruments et par l’embryotomie. Les symphysiotomies ou encore l’accouchement prématuré provoqué prônés par des anglo-saxons, étaient indiqués en cas de rétrécissement pelvien. L’absence totale d’asepsie et de suture utérine entraînaient une mortalité tellement sévère (entre 50% et 90%) que la CS était pratiquée exclusivement en cas de dystocie pelvienne insurmontable.

Depuis, la technique opératoire de la césarienne a connu une évolution spectaculaire. L’avènement de la césarienne segmentaire imposée par Brindeau en France en 1921 fut certainement le progrès le plus décisif de l’histoire de la CS. L’amélioration progressive du pronostic maternel fut étroitement liée non seulement aux progrès de la technique opératoire mais aussi aux innovations de l’anesthésie, à l’introduction de la notion d’asepsie, à l’avènement des antibiotiques, de la transfusion sanguine et la lutte contre les accidents thromboemboliques. En définitif, toutes ces avancées médicales ont contribué à la transformation de la CS primitive toujours tragique en une intervention maîtrisée et sûre.

Au cours des années 1950, alors que les CS “segmentaires” étaient déjà pratiquées, l’intervention durait environ une heure et les pertes sanguines devaient habituellement être compensées. Par la suite et au cours des dernières décennies du 20^ème^ siècle, d’autres améliorations des techniques chirurgicales obstétricales basées sur des concepts modernes et scientifiquement fondés ont été réalisées et ont conduit à une approche plus simple de la CS . Ainsi, en 1999, Stark a introduit la technique Misgav Ladach (MLC) . L’objectif de Stark était de proposer une technique de CS sûre, simple, rapide et facilement reproductible quelles que soient les conditions opératoires. Avec la MLC, environ trente minutes suffisent pour une CS et la perte sanguine moyenne est à peu près la même que dans le cas d’un accouchement par les voies naturelles .

Cependant, depuis la fin des années 90, les techniques de CS n’ont pas connu de progrès important. La méthode MLC, décrite il y a plus de 20 ans, reste la technique la plus couramment utilisée dans le monde et ce, jusqu’à nos jours .

Plus récemment, avec le concept de soins périnataux centrés sur la femme, le retour aux approches extra-péritonéales est de plus en plus défendu en raison de l'amélioration de la réhabilitation post-opératoire et des objectifs de préservation de la fertilité future.

La French AmbUlatory C section (FAUCS) décrite par Denis Fauck et Jacques Henri Ravina est bien différente de toutes ces techniques réunies, car elle combine un nombre important d’innovations comme la voie d’abord et la méthode de suture utérine .

Dans une étude rétrospective portant sur plus de 3000 cas, la FAUCS semble permettre un temps de convalescence plus court, avec une sortie de l'hôpital le lendemain de la chirurgie dans 90% des cas. Nous avons introduit la FAUCS pour la première fois en Tunisie le 12 Janvier 2018. À ce jour, il n’existe aucune étude prospective comparative qui évalue les résultats de la FAUCS avec un niveau de preuve scientifique élevé.

Dans ce sens, nous avons mené ce travail. Notre objectif principal était de tester l’hypothèse selon laquelle la FAUCS réduit la douleur postopératoire sans augmenter la morbidité maternelle et néonatale.

1. **Cadre et type de l’étude**

Notre étude sera menée au service de gynécologie obstétrique, du centre hospitalo-universitaire Mongi Slim, La Marsa.

Il s’agira d’une étude interventionnelle, prospective, randomisée et contrôlée.

L’intervention est de type chirurgical. En effet, nous comparerons deux techniques chirurgicales de césarienne :

- La technique Misgav Ladach (MLC).

- La French AmbulatoryCesarean Section » (FAUCS) . Cette méthode est détaillée dans l’annexe 1. Pour la fermeture cutanée, nous avons opté pour un surjet intradermique au lieu de la colle Dermabond® pour que les patientes soient indiscernables en apparence.

Nous avons défini deux groupes de patientes :

- Groupe MLC : Groupe défini par les patientes ayant reçu l’intervention : césarienne programmée selon méthode MLC.

- Groupe FAUCS : Groupe défini par les patientes ayant reçu l’intervention : césarienne programmée selon la méthode FAUCS.

Nous avons respecté les règles CONSORT 2010 pour l’élaboration du protocole de l’étude [9].

L’étude sera enregistrée sur le site Clinical Trials. gov après accord du comité d’éthique

La première patiente sera recrutée en Aout 2018.

1. **Population de l’étude**

L’étude portera sur l'ensemble des femmes qui ont accouché à terme par une césarienne programmée en dehors du travail dans le service de gynécologie obstétrique du centre hospitalo-universitaire de Mongi Slim La Marsa pendant la période concernée.

1. **Les Critères d’éligibilité**
   1. **Critères d’inclusion**

Nous inclurons toutes les parturientes répondant aux critères suivants :

- Âge supérieur à 18 ans et inferieur à 48 ans .

- Grossesse monofoetale.

- Age gestationnel > ou égal à 37 SA .

- Indication obstétricale d’un accouchement par une césarienne programmée comme:

*Utérus cicatriciel et contre-indication à l'épreuve utérine (utérus bi ou multi cicatriciel, cicatrice utérine corporéale, myomectomie intra-murale, bassins chirurgicaux ou rétrécis, présentation dystocique).

*Présentation du siège et contre-indication à la voie basse.

*Macrosomie fœtale avec un poids estimé supérieur ou égal à 4Kg.

*Placenta prævia.

- 1. **Critères de non inclusion**

Dans notre étude, nous n’allons pas inclus les parturientes présentant au moins un des critères suivants :

- Une pathologie fœtale connue en prénatal : retard de croissance sévère avec anomalies Doppler, malformation, anomalie génétique ...

- Une anomalie d’adhésion placentaire.

- Un accouchement par une césarienne au cours du travail, dans un contexte d'urgence médico-obstétricale.

- 1. **Critères d’exclusion**

Dans notre étude, nous excluront les parturientes répondant à au moins un des critères suivants :

- Absence de consentement.

- Patientes avec une ancienne cicatrice cutanée verticale.

- Les patientes qui seront initialement recrutées et programmées pour un accouchement par une césarienne en dehors du travail mais qui auront dû être opérées soit avant la date prévue initialement soit par un opérateur autre que ceux assignés pour l’étude.

- Les patientes qui nécessiteront une conversion en anesthésie générale avant l’extraction fœtale suite à un échec de l’anesthésie locorégionale.

1. **Critères de jugement**
   1. **Critère de jugement principal**

Le principal critère de jugement dans notre étude sera le score de douleur postopératoire moyen (SDPM).

En effet, la douleur postopératoire sera évaluée au moyen de l’échelle visuelle analogique (EVA) [10] et ce, régulièrement au cours du premier jour post opératoire à savoir à H0, H6, H12, H18 et H24. Le SDPM a été défini comme suit :

SDPM = (EVA H0 + EVA H6 + EVA H12 + EVA H18 + EVA H 24) /5.

- 1. **Critères de jugement secondaires**

Les critères de jugement secondaires dans notre étude seront classés comme suit :

- - 1. **Critères relatifs au déroulement de la chirurgie**
       1. **Temps opératoires**

- T1: défini par le temps écoulé entre l'incision cutanée et l’hystérotomie et exprimé en minutes.

- T2: défini par le temps écoulé entre l’hystérotomie et l’extraction fœtale et exprimé en minutes.

- T3: défini par la durée en minutes de l’hystérorraphie.

- T4: défini par la durée totale de l’intervention et exprimé en minutes.

- - - 1. **Complications per opératoires**

Nous relèveront dans ce sens la survenue éventuelle des complications suivantes: plaie vésicale ou urétrale, plaie vasculaire (pédicules utérins ou vaisseaux iliaques), trait de refend sur le segment inférieur, plaie digestive ...

- - - 1. **Pertes sanguines estimées visuellement (PSEV)**

Cette estimation tiendra principalement compte du volume du liquide aspiré en per opératoire dans le bocal gradué et exprimé en ml.

- - - 1. **Pertes sanguines totales calculées (PSTC)**

Le calcul des pertes sanguines tiendra compte du volume sanguin total (VST), de l’hématocrite prépartum, de l’hématocrite post-partum, selon les formules suivantes :

- PSTC = VST * Le pourcentage de pertes sanguines

- VST = 0,75 x {[taille (en pouces) x 50] + [poids (en livres) x 25]}

- Le pourcentage de pertes sanguines = ({hématocrite pré-partum – hématocrite post-partum}/ hématocrite pré-partum

- - 1. **Critères relatifs à l’issue néonatale**

Nous évalueront l’état du nouveau-né par :

- Le score d’Apgar.

- L’équilibre acido-basique néonatal :

* Valeurs du pH au niveau de l’artère ombilicale (pH), de la pression CO2 (pCO2)

* Valeur du pH corrigé sur la capnie : pH eucapnique .

- - 1. **Critères relatifs à l’autonomie postopératoire des patientes**
       1. **Douleur post-opératoire**

Elle sera évaluée par :

- Les scores EVA durant le premier jour post opératoire : EVA H0, EVA H6, EVA H12, EVA 18, EVA 24.

- Le maximum de la douleur ressentie durant le premier jour : EVA max.

- La voie d’administration, la dose (mg) et le palier des antalgiques prescrits.

- - - 1. **Délai au premier lever**

Ce délai, exprimé en heures, est défini par le temps qui sépare la fin de l’acte opératoire et le moment où la patiente a pu se lever toute seule de son lit pour la première fois.

- - - 1. **Délai au premier repas complet**

Ce délai, exprimé en heures, est défini par le temps qui sépare la fin de l’intervention et la reprise d’une alimentation orale normale (solide + liquide).

- - - 1. **Durée du séjour postopératoire**

Ce délai, exprimé en jours, est défini par le temps séparant la fin de l’acte opératoire et la date de retour à domicile.

1. **Taille de l’échantillon**

Nous avons calculé la taille de l’échantillon en considérant le score de douleur postopératoire moyen (SDPM) comme critère de jugement principal.

Notre objectif était de mettre en évidence une diminution du SDPM de 60% entre les deux groupes tout en tenant compte du niveau de signifiance (p=0,05), de la puissance attendue de l’étude (80%), et des valeurs de α = 0,05 et β = 0,10.

Ainsi, nous avons utilisé la formule suivante :

N = M / DS^2^ ; M = 2 * (z1-α + z1-β); DS = (m1 – m2)/ σ

m1= SDPM dans le groupe MLC, m2 = SDPM dans le groupe FAUCS et σ = écart type de m1.

La moyenne (m1) et l'écart type (σ) du score douleur dans le groupe MLC ont été estimés à partir des dossiers de 80 patientes opérées par cette technique en 2017.

Ces valeurs étaient approximativement m1 =3,8 ; σ = 2.

Ainsi, nous avons obtenu qu’un minimum de 45 patientes était nécessaire dans chaque groupe. En tenant compte de la possibilité de perte de vue de 10% des patientes, nous avons recruté 50 dans chaque groupe.

1. **Déroulement de l’étude**
   1. **Visite de pré-inclusion**

Toute parturiente éligible et suivie à la consultation externe de notre service sera informée de l’étude (objectifs, déroulement et bénéfices attendus) au cours de la visite prénatale du 8^ème^ mois.

Un délai de réflexion de 7 jours lui sera accordé pour la signature du formulaire de consentement .

Une liste préliminaire des femmes pré-incluses serapar un investigateur n’ayant aucune intervention directe dans le protocole.

- 1. **Inclusion**

L’inclusion sera finalisée le jour programmé de l’accouchement. En effet, parmi les parturientes initialement pré incluses, certaines peuvent accoucher en dehors de la date prévue de l’accouchement ou encore à la date prévue mais sans que les opérateurs sollicités pour l’étude ne soient disponibles (cf: critères d’exclusion). Ainsi, nous avons opté pour retarder l’attribution des numéros au jour de la césarienne programmée et au patientes ayant dépassé tous les critères d’exclusion.

De la sorte, le jour même de la césarienne programmée et après inclusion finale des parturientes à l’étude, nous leur chronologiquement et respectivement attribué un numéro de 1 à 100.

- 1. **Technique de randomisation**

La répartition des femmes retenues pour l’étude est basée sur la randomisation selon la méthode de la Table de KENDALL et B.B. SMITH .

Nous avons fait correspondre les numéros d’ordre attribués lors de l’inclusion aux numéros prélevés à partir de la table de nombre au hasard assignant chacune des femmes incluses soit au groupe MLC soit au groupe FAUCS .



- 1. **L’aveugle**

Les patientes incluses dans l’étude n’auront pas connaissance de la méthode chirurgicale. Elles seront informées de la technique utilisée le jour de leur sortie de l'hôpital.

Les deux techniques seront identiques en apparence. Les patientes des deux groupes seront donc indiscernables. De même, tous les intervenants dans la prise en charge pré ou post opératoire (infirmières, sages-femmes, aide soignantes, internes, résidents …) n’auront pas connaissance du type de chirurgie réalisé.

Le chirurgien et l’anesthésiste seront informés de la technique à réaliser juste avant de démarrer l’acte, dans la salle opératoire. Ils n’auront pas non plus connaissance du contenu de la liste finale des parturientes ou des numéros attribués le jour de la césarienne. En effet, seul l’investigateur responsable de l’échantillonnage aura connaissance de la chronologie de l’avancement de l’étude.

- 1. **Déroulement de l’intervention**
     1. **En pré-opératoire**

Une consultation pré-anesthésique et une réserve de deux poches de sang seront obligatoires pour toutes les patientes. La fiche de pré-anesthésie, la comptabilité et la validité des poches de sang sera vérifiés par l’anesthésiste avant l’entrée de la patiente au bloc opératoire.

Une numération et formule sanguine préopératoire sera systématiquement réalisée pour toutes les patientes de l’étude.

Une miction sera faite par toutes les patientes avant le passage au bloc opératoire. Pour le groupe MLC, le sondage vésical sera réalisé par le chirurgien après l’anesthésie locorégionale. Pour le groupe FAUCS, le sondage vésical ne sera pas nécessaire.

Le recueil des données en pré-opératoire sera assuré par l’interne affecté à la salle de travail selon une fiche individuelle d’évaluation (Annexe 4). Ainsi, il rapportera toutes les caractéristiques épidémiologiques de la patiente (poids, taille, âge, parité …), les caractéristiques de la grossesse en cours (terme gestationnel, dysgravidie, indication de la césarienne…)

- - 1. **En per-opératoire**
       1. **Les opérateurs**

Les opérateurs assignés pour le groupe FAUCS seront deux chirurgiens séniors préalablement formés à cette technique avec un nombre moyen de 50 actes réalisés avant le démarrage de l’étude.

Les opérateurs assignés pour le groupe MLC seront soient les mêmes seniors (en fonction de leur disponibilité) ou les résidents majors ayant l’expérience de la méthode MLC avec un nombre moyen de 50 actes réalisés avant le lancement de l’étude.

- - - 1. **L’anesthésie**

Le médecin anesthésiste sera informé de la technique opératoire le jour de l’acte. Toutes les patientes de l’étude auront une anesthésie locorégionale. Le protocole d’anesthésie ne sera pas le même dans les deux groupes. En effet, dans le groupe FAUCS, l’anesthésie locorégionale ne contient pas de morphine. Les protocoles d’anesthésie des deux groupes de l’étude sont détaillés dans le tableau I.

**Tableau N° I :** Protocoles d’anesthésie adoptés dans les deux groupes de patientes.

| **GROUPE** | | **FAUCS** | **MLC** |
| --- | --- | --- | --- |
| Sonde vésicale | | Non | Oui |
| Remplissage | | Non | Oui (500 millilitres de  sérum physiologique) |
| Anesthésie  locorégionale | Marcaine | 7 à 9 mg  (selon de la taille de la patiente) | 8 à 10 mg  (selon de la taille de la patiente) |
|  | Fentanyl | 10 gamma | 10 gamma |
|  | Morphine | Non | 100 gamma |

- - - 1. **La chirurgie**

Le tableau II résume les points de différence entre les deux techniques chirurgicales comparées dans cette étude.

**Tableau N° II :** Techniques chirurgicales respectivement adoptées dans les deux groupes de patientes

| **GROUPE** | **FAUCS** | **MLC** |
| --- | --- | --- |
| Incision cutanée | Horizontale  au niveau du pli  abdominal inférieur | Horizontale  à 3 cm au-dessous de la ligne inter iliaque unissant les deux épines iliaques antéro-postérieures |
| Incision de l’aponévrose | Verticale  para-médiane gauche | Horizontale  au même niveau que l’incision cutanée |
| Abord du segment inférieur de l’utérus | Extrapéritonéal  latéro-vésical gauche | Intrapéritonéal |
| Hystérotomie | Segmentaire,  arciforme et transversale | Segmentaire,  Arciforme et transversale |
| Extraction fœtale | Recours aux instruments systématique en cas de présentation céphalique  (instrument utilisé : forceps Wrigley®) | Recours aux instruments uniquement en cas de difficultés peropératoires |
| Expression sur le fond utérin lors de l’extraction fœtale | Non | Systématique |
| Délivrance | Dirigée  par 5UI ocytocine IV après accouchement de l'épaule. | Dirigée  par 5UI ocytocine IV après accouchement de l'épaule. |
| Extériorisation de l’utérus | Jamais | Au besoin |
| Hystérorraphie | En bourse | Surjet simple |
| Fermeture de l’aponévrose | Surjet simple  de fil résorbable  (Neofil 1®) | Surjet simple  de fil résorbable  (Neofil 1®) |
| Fermeture plan sous cutané | Surjet simple  de fil résorbable  (Neofil 1® rapide) | Surjet simple  de fil résorbable  (Neofil 1® rapide) |
| fermeture cutanée | Surjet intradermique  de fil résorbable  (Neofil ® rapide) | Surjet intradermique  de fil résorbable  (Neofil ® rapide) |

Chez toutes les parturientes de l’étude, dès l’extraction fœtale, un prélèvement sanguin sur l’artère ombilicale sera réalisé. Le cordon ombilical sera clampé immédiatement, avant le premier cri de l’enfant, par deux pinces. La ponction sera faite avec une seringue de 10 ml héparinée au niveau de l’artère ombilicale en évitant les bulles d’air.

Le recueil des données per-opératoires sera assuré par l’interne du bloc opératoire selon une fiche individuelle d’évaluation (Annexe 4). Ainsi, il notera prospectivement les différents temps opératoires, les modalités de l’extraction fœtale, les résultats de l’étude de l’équilibre acido-basique néonatal, les pertes sanguines évaluées visuellement (PSEV) …

- - 1. **Le post-opératoire**

Aucun membre de l’équipe prenant en charge les patientes en post-opératoire n’aura pas connaissance de la technique chirurgicale réalisée.

La surveillance pendant les deux premières heures sera assurée en unité post-opératoire immédiat par l’opérateur et l’anesthésiste. Ces derniers releveront les constantes hémodynamiques, l’abondance des saignements et la qualité du globe utérin.

Pour la prévention de l’hémorragie du postpartum, toutes les patientes recevront une injection intraveineuse lente de 5 à 10 UI d’oxytocine (Syntocinon^®^) dans 500 ml de Ringer Lactate ou de sérum glucosé.

Chez les patientes stables, le transfert de la patiente à l’unité post-opératoire du service sera indiqué à la fin de la deuxième heure (H2). La surveillance ultérieure sera assurée par l’interne affecté à l’unité post-opératoire du service ainsi que par les internes de garde selon les mêmes modalités précédemment décrites. De plus, ces derniers et sans avoir connaissance de la technique chirurgicale adoptée auront pour mission de calculer les scores EVA toutes les six heures durant les premières 24 heures.

La prise en charge post-opératoire sera similaire dans les deux groupes de patientes. Ainsi, une numération formule sanguine postopératoire sera systématiquement réalisée. Un lever était systématiquement proposé par l’infirmière dès la première heure post opératoire et puis toutes les heures jusqu’à ce que la patiente se sente apte à se lever.

Dans le groupe MLC, la sonde vésicale sera retirée dès le premier lever.

La reprise de l‘alimentation orale sera autorisée dès l’émission des gaz dans les deux groupes.

La prise en charge de la douleur post-opératoire repondera à un schéma unique pour toutes les patientes de l’étude. La prescription d’antalgiques ne sera pas systématique mais guidée principalement par la demande des patientes et l’évaluation de l’intensité de la douleur avec l’EVA.

Le schéma adopté sera le suivant :

- En première intention : 100 mg de kétoprofène en intra rectal toutes les 6 heures.

- En deuxième intention : 1 gramme de paracétamol par voie veineuse toutes les 6 heures.

- En troisième intention : 50 milligrammes de tramadol per os toutes les 6 heures.

Le recueil des données post-opératoires sera assuré par l’interne affecté à l’unité post opératoire selon une fiche individuelle d’évaluation . Ce dernier n’avait pas accès aux comptes rendus opératoire. De même, les chirurgiens et l’anesthésiste n’auront pas accès aux données du suivi post-opératoire.

1. **Etude statistique**
   1. **Variables étudiées**

Nous compareront les deux groupes de l’étude en étudiant des variables à la fois quantitatives et qualitatives réparties en quatre catégories:

-Variables relatives aux Caractéristiques épidémiologiques des patientes :

Âge, indice de masse corporelle, parité, nombre d’accouchement par voie basse et nombre total de cicatrices utérines.

-Variables relatives au déroulement de la grossesse:

Dysgravidies, terme gestationnel, indication de la césarienne.

-Variables relatives à la technique chirurgicale:

T1, T2, T3, T4, les pertes sanguines (PSEV, PSTC), modalités et durée de l’extraction, difficultés rapportées par l’opérateur et complications per-opératoires.

Dr-Variables relatives à l’issue néonatale:

Scores d’Apgar, pH au cordon, pH eucapnique, poids à la naissance, nombre et motifs d’hospitalisation en néonatologie.

-Variables relatives au post-opératoire: Scores EVA, SDPM, EVA max, paliers des antalgiques prescrits, dose totale d’antalgiques nécessitée au cours des premières 24 heures, délai au premier lever, délai au premier repas complet, complications post opératoires et durée du séjour à l’hôpital.

- 1. **Analyse statistique**

Toutes les analyses seront réalisées au moyen du logiciel RStudio (version 3.5.2).

Nous analyseront la distribution des variables de l’étude par le test de normalité shapiro-wilk.

Les variables quantitatives seront analysées et exprimées en moyennes [min-max] ou médianes [1er-3ème quartiles]. La comparaison utilisera le test t de Student en cas de distribution normale et le test de Mann-Withney en l’absence de distribution normale.

Les variables qualitatives seront exprimées en pourcentages. La comparaison entre les deux groupes utilisera le test du χ².

Le seuil de significativité adopté sera de 0,05.

Pour visualiser les résultats, nous utiliseront plusieurs types de diagrammes: le diagramme en bâtons, le diagramme circulaire (camembert), le diagramme en boîte à moustaches, les histogrammes et les courbes de densité de Kernel [17].

1. **Recherche bibliographique**

La recherche bibliographique en anglais a été réalisée sur le moteur de recherche Pub-Med en utilisant le thésaurus MeSH. Les mots clés utilisés étaient : Cesarean section, Misgav Ladach method, Maternal outcome, extra-peritoneal approach, fœtal outcome, postoperative pain.

La recherche bibliographique en français a été réalisée sur le moteur de recherche EMC consulte.

Les mots clés utilisés étaient : césarienne, extraction instrumentale, douleur post-opératoire, pertes sanguines, autonomie post-opératoire, césarienne extra- péritonéale.

1. **Conflits d'intérêt**

Nous déclarons n’avoir aucun conflit d’intérêt.

Le consentement écrit sera obtenu chez toutes les patientes . Nous respecteront la loi tunisienne concernant la protection des données personnelles.

**Dr Kaouther Dimassi**

**Professeur Agrégé**

**Service de gynecologie obstétrique**

**Hopital universitaire Mongi Slim La Marsa**

**COPIE CONSENTEMENT EN ARABE**

إني الممضية اسفله --------------------------------------- (اسم ولقب المريضة)، أوافق على المشاركة في دراسة تقارن بين طريقتين للولادة القيصرية : تقنية "كوان سارك " والتقنية الفرنسية"فوكس"

حصلت على شرح مفصل عن الدراسة وأهدافها وإجراءاتها من قبل الدكتور .................. ............. (اسم ولقب الطبيب)

في حال وافقت على المشاركة في هذه الدراسة، سيبقى إسمي طي الكتمان . لن يكون لأي شخص، ما لم ينص القانون على ذلك، حق الإطلاع على ملفي الطبي باستثناء الطبيب المسؤول عن الدراسة ومعاونيه، ولجان الأخلاق المهنية المستقلة

إذا تم نشر نتائج هذه الدراسة ، ستبقى البيانات سرية

إن مشاركتي في الدراسة طوعية

أنا حرة في قبول أو رفض المشاركة. هذا لن يؤثر على جودة الرعاية التي سيتم تقديمها لي

الدراسةمنلانسحابقررتحالمعطياتيإتلافعلىالاشرافبامكاني

إن موافقتي لا تعفي المشرفين على هذه الدراسة من مسؤولياتهم. أحتفظ بجميع حقوقي التي يضمنها القانون

فهمت كل المعلومات التي قدمت الي وتلقيت إجابة على كل أسئلتي و أوافق على أن أشارك في هذه الدراسة بطوعية وبدون أي نوع من الاجبار أو الضغوط

اسم ولقب المريضةاسم ولقب الطبيب

الامضاءالامضاء

**COPIE CONSENTEMENT EN FRANÇAIS**

Je soussigné(e) …………………………………………………………(nom et prénom de la patiente), accepte de participer à l’étude comparative de deux techniques de césarienne: la césarienne selon la technique Cohen-Stark et la césarienne extrapéritonéale selon la technique « French AmbulatoryCesarean Section » (FAUCS).

Les objectifs et modalités de l’étude m’ont été clairement expliqués par le Dr……………….............(nom et prénom du médecin).

J’accepte que les documents de mon dossier médical qui se rapportent à l’étude puissent être accessibles aux responsables de l’étude et éventuellement aux autorités de santé. A l’exception de ces personnes, qui traiteront les informations dans le plus strict respect du secret médical, mon anonymat sera préservé.

Si les résultats de cette étude seront publiés, les données resteront anonymes.

J’ai bien compris que ma participation à l’étude est volontaire.

Je suis libre d’accepter ou de refuser de participer. Cela n’influencera pas la qualité des soins qui me seront prodigués.

J’ai le droit de demander et à assister à la destruction de mes données personnelles si je décide de ne plus faire partie de l’étude.

Mon consentement ne décharge pas les organisateurs de cette étude de leurs responsabilités. Je conserve tous mes droits garantis par la loi.

Après en avoir discuté et avoir obtenu la réponse à toutes mes questions, j’accepte librement et volontairement de participer à l'étude qui m’est proposée.

Nom de la patiente : Nom de l'investigateur :

Signature: Signature:
