# Supplementary material for: The extraperitoneal French AmbUlatory cesarean section technique leads to improved pain scores and a faster maternal autonomy compared with the intraperitoneal Misgav Ladach technique: A prospective randomized controlled trial
Source: PLoS One. 2021 Jan 22;16(1):e0245645. doi: 10.1371/journal.pone.0245645 (PMC7822305; doi:10.1371/journal.pone.0245645)
Supplement: S2 File — (PDF) [file pone.0245645.s004.pdf]

Study identification : NCT03741907

TiTle : Comparing French Ambulatory and MISGAV-LADACH C-Section Techniques

Principal investigator : Professor kaouther dimassi

kaouther.dimassi@fmt.utm.tn

Location : Mongi Slim University Hospital , La Marsa , TUNISIA

Date of approval : 05.05.2018

STUDY PROTOCOL

This study is a randomized clinical trial conducted at Mongi Slim University Hospital, La Marsa, Tunisia; between August 2018 and January 2019.

The study was approved by the local hospital ethics committee (reference number 05/18) and was registered at clinicaltrials.gov (NCT03741907). Data collection was conducted in compliance with Tunisian laws regarding personal data protection.

During the study period, women with a singleton pregnancy and an elective CS indication were eligible. There were no restrictions regarding the number of previous CS, fetal presentation or fetal weight. Following subjects were not included in the sample: women under 37 weeks of gestation, women with a prenatally diagnosed fetal pathology (intrauterine growth restriction, malformation, genetics disorders), a morbidity adherent placenta, an adnexal mass or a myoma in the lower uterine segment.

All women who met the inclusion criteria were invited to participate in the study during their final prenatal visit. Those providing written informed consent were consecutively included in a preliminary patient list managed by an investigator not involved in patient care.

Before randomization, we excluded women who were initially recruited but who had to be operated either as an emergency before the originally scheduled date (example: in case of acute fetal compromise) or by a different surgeon than those assigned to the study (Figure

1). Women recruited into the study were given a study number on theirdelivery day, ina chronological order.

All participants were randomly assigned to either FAUCS or MLC group. Group allocation was predetermined by an investigator who was not involved in patients care and using the Kendall B.B Smith table [7].

The participants as well as, the residents involved in patient care, and the investigators were blinded to the cesarean technique before the operation and were informed at discharge. Both anesthetists and surgeons were informed of the cesarean technique on the due date in the operating room.

The MLC spinal anesthesia protocol included 500cc of isotonic crystalloid solution for vascular filling, bupivacaine 7 to 10 milligrams (depending on the patient's height), morphine 100 micrograms, and sufentanil 10 micrograms.

The FAUCS spinal anesthesia protocol included no vascular filling, no morphine, bupivacaine 7 to 10 mg (depending on the patient's height), and sufentanil 10 micrograms.

A bladder catheterization was performed on the MLC group by using a 16 or 18-F catheter prior to the skin incision. In both groups, a single dose of a first-generation cephalosporin was administered intravenously.

All FAUCS surgeries were performed by two senior surgeons (K.D and A.H). Both were experienced with the paravesical approach and had performed more than 30 cases previously. The MLC cesarean operations were performed by same seniors or residents who performed vast majority of the operations in the unit.

For this study, we performed the MLC technique as described in 1999 [3].Regarding the FAUCS approach described in 2017, the use of skin glue for closure is recommended [6]. In order to

ensure that all the patients were virtually indistinguishable, we alternatively employed a subcuticular absorbable Vicryl suture for the skin closure.

Hematocrit levels were assessed before and after surgery. Analgesics were administered by the nursing staff, upon patient's requests and visual analogue scale (VAS) [8] . Assessments with VAS were made every 6 hours: H0,H6,H12,H18, and H24. A standardized analgesics scheme was used:

First intention: 100 mg Ketoprofen intra-rectal route every 6 hours.

Second intention: intra venous1g paracetamol every 6 hours.

Third intention: tramadol 50 mg oral route every 6 hours.

A first standing was proposed to all women everyhour during the first post operative day.

The bladder catheter was removed as soon as the woman was able to walk alone.Normal oral food intake was initiated as soon as gas passage occurred and the patient felt hungry.

Women were evaluated by the end of the first day. If there were no complications and if the patient felt autonomous and pain-free, they were discharged 24 hours after the surgery. In the other cases, patients were discharged at least 48 hours after surgery after a similar evaluation of maternal autonomy.

The primary outcome measure of the study was the post-operative mean pain score (PMPS). PPMS = (VAS H0+ VAS H6 + VAS H12+ VAS H18 + VAS H24)/5.

Secondary Outcome Measures were:

- VAS max : defined as the highest post operative pain score felt during the first day.
- Total dose of administrated analgesics during the first 24 hours (mg).

- Calculated estimation of blood loss (cEBL): derived by multiplying the calculated maternal blood volume by the percent of blood volume lost, were the maternal blood volume was calculated by Nadler's formula based on the patient's weight, and height. The percent of blood volume lost was calculated with Brecher's formula[9] [{predelivery hematocrit (HCT) – post delivery HCT}/predelivery HCT)].
- Surgery time in minutes: T1: Delay between skin incision and hysterotomy; T2: Delay between hysterotomy and fetal extraction (min); T3: time spent for uterine sutures; T4: total duration of the surgery.
- Rate of short-term incidents: hemorrhage, transfusion, bladder injury or any other complication.
- Maternal autonomy: time to first spontaneous urination, time to first standing; time to first complete meal.
- Newborn overall condition: Neonatal acid base balance (Cord blood gases, pH and eucapnic pH[10]; Apgar score at 1 and 5 minutes after birth, rate of neonate hospitalization.
- Delay to hospital discharge (days).

Statistics

The sample size (N) calculations were based on the post-operative mean pain score (PMPS) as the main outcome measure. The mean (m_1) and standard deviation (σ) for the PPMS in the MLC group were estimated by reviewing the records of 80 consecutive women who underwent this procedure in 2017.These values were approximately m1=3,8; σ=2. We aimed to detect a decrease of 60% in the PMPS in the FAUCS group (m_2), taking into account the level of significance (p=.05) and the expected power of the study (80%), with α=0.05 and β=0.10; we used the following formula: $N = M / D_s^{2}$; $M = 2 * (z_{1-\alpha} + z_{1-\beta})=17.1$; $D_s = (m_1 - m_2)/ \sigma$; $N= 17.1/ (0.6\, m1/\sigma)^{2}$. 45 patients per group were required to detect a

decrease of 60% in PMPS scores between the two groups. Ultimately, we recruited 50 participants for each group to account for possible losses.

We measured 44 outcome variables. The metrics were grouped into four categories: surgery, overall morbidity, neonatal outcomes and maternal autonomy during recovery. All the analyses were conducted in RStudio (version 3.5.1). We performed unequal variance t-tests for all continuous variables and evaluated each distribution for normality using the Shapiro Wilk test. Continuous variables were analyzed with the student's t test and Mann-Whitney test. We performed asymptotic generalized Pearson's chi-squared tests for the categorical variables. When the categorical variables were ordinal, we used the Cochran-Armitage test, comparing one ordinal variable with a dichotomous one. All tests were performed as 2-tailed tests, and $p<.05$ was accepted as statistically significant.

To visualize the results, we employed smoothed kernel density plots of continuous outcome variables for FAUCS compared with the MLC values, which were viewed on a log scale where applicable.

1. Dodd JM, Anderson ER, Gates S, Grivell RM. Surgical techniques for uterine incision and uterine closure at the time of caesarean section. Cochrane Database Syst Rev. 2014 Jul 22;(7):CD004732.

2. Kulas T, Bursac D, Zegarac Z, Planinic-Rados G, Hrgovic Z. New Views on Cesarean Section, its Possible Complications and Long-Term Consequences for Children's Health. Med Arch. 2013 Dec;67(6):460–3.

3. Katsulov A, Nedialkov K, Koleva Z, Iankov M, Tashkov B, Iotov T, et al. [The Joel-Cohen (MisgavLadach) method--a new surgical technic for cesarean section and gynecological laparotomy]. AkushGinekol (Sofiia). 2000;39(1):10–3.

4. Tappauf C, Schest E, Reif P, Lang U, Tamussino K, Schoell W. Extraperitoneal versus transperitoneal cesarean section: a prospective randomized comparison of surgical morbidity. Am J Obstet Gynecol. 2013 Oct;209(4):338.e1-8.

5. S BD, J C. Extraperitoneal versus transperitoneal cesarean section in surgical morbidity in a tertiary care centre. Int J ReprodContraceptObstet Gynecol. 2017 Jul 26;6(8):3397–9.

6. Olivier A, Mathieu F, Bénédicte S, Richard B, Jean-Jacques C, Luka V, et al. The French Ambulatory Cesarean Section: Technique and Interest. Int J GynecolClinPract [Internet]. 2017 Aug 4 [cited 2019 Apr 21];2017. Available from: https://www.graphyonline.com/archives/IJGCP/2017/IJGCP-131/index.php?page=abstract

7. Kendall MG, Smith BB. Tables of random sampling numbers [Internet]. Cambridge [England] : U.P; 1961 [cited 2019 Apr 21]. Available from: https://trove.nla.gov.au/version/27753808
