# Supplementary material for: The extraperitoneal French AmbUlatory cesarean section technique leads to improved pain scores and a faster maternal autonomy compared with the intraperitoneal Misgav Ladach technique: A prospective randomized controlled trial
Source: PLoS One. 2021 Jan 22;16(1):e0245645. doi: 10.1371/journal.pone.0245645 (PMC7822305; doi:10.1371/journal.pone.0245645)
Supplement: Attachment — Submitted filename: response to comments .docx [file pone.0245645.s005.docx]

ONE-D-20-25216
The extraperitoneal French AmbUlatory cesarean section technique leads to superior maternal outcomes compared with the intraperitoneal Misgav Ladach technique: A prospective randomized controlled trial
PLOS ONE

Dear Dr. Kaouther Dimassi
Thank you for submitting your manuscript to PLOS ONE.

I am sending the manuscript back to you as I would like need some clarifications to be considered.

The trial started in August 2018, however the trial registration was done in November 15, 2018. In general a trial must be registered prior start of any patient enrolment. Could you please state the discrepancy in your submission letter?

 This decrepency was added to the cover letter with this citation :

This study was approved by the local hospital ethics committee before the start of enrollment on May 5, 2018 (reference number 05/18) and was registered at Clinicaltrials.gov (NCT03741907) in november 2018 . Data collection was conducted in compliance with Tunisian laws regarding personal data protection.

The method section is missing several mandatory aspects of a clinical trial including approach to randomization or sequence generation and /or block size used.

 The two comparison groups were generated using simple randomization, with an equal allocation ratio, by referring to Kendall and Smith’s Tables of Random Sampling Numbers .

This information was added in the methods section as recommended

The inclusion/exclusion criteria in the trial registration and the manuscript are not identical.

 maybe no identical bu synonyms . we detailed the inclusion and exclusion criteria in order to fit perfectly to the protocol

Women were included if they had a singleton term pregnancy with an indication of a planned CS. There were no restrictions regarding the number of previous CS, fetal presentation or fetal weight. Women were excluded if their pregnancies involved known fetal, placental, or uterine anomalies ( intrauterine growth restriction, malformations, genetic disorders, adnexal mass, myoma in the lower uterine segment, morbidity adherent placenta …).

The clinical trial registration states 100 patients while your sample size only states 90.

 Sample size calculation was based on the PMPS. The effect of FAUCS was considered important if the difference in PMPS between groups was ≥ 30%. To achieve 80% power with an α = 0.05, it was anticipated that at least 45 patients in each group were required.     Taking into account the risk of loss to follow up we recruited 50 patients in each group.

This information was added to the statistical analysis section

Was the statistical analysis done as per intention to treat, as this was not stated in the statistical section?

 Yes information added

If you could edit this in the manuscript and resubmit afterwards, it would be appreciated, I will get your manuscript out for peer-review.

Yours sincerely,

Georg M. Schmölzer
Academic Editor
PLOS ONE


[Note: HTML markup is below. Please do not edit.]
