# Supplementary material for: The extraperitoneal French AmbUlatory cesarean section technique leads to improved pain scores and a faster maternal autonomy compared with the intraperitoneal Misgav Ladach technique: A prospective randomized controlled trial
Source: PLoS One. 2021 Jan 22;16(1):e0245645. doi: 10.1371/journal.pone.0245645 (PMC7822305; doi:10.1371/journal.pone.0245645)
Supplement: Attachment — Submitted filename: Response_to_Reviewers.docx [file pone.0245645.s006.docx]

If you will need more time than this to complete your revisions, please reply to this message or contact the journal office at [plosone@plos.org](mailto:plosone@plos.org). When you're ready to submit your revision, log on to <https://www.editorialmanager.com/pone/> and select the 'Submissions Needing Revision' folder to locate your manuscript file.

Georg M. Schmölzer

Academic Editor

PLOS ONE

Object : PONE-D-20-27208 - Rebutal letter

Dear Editor,

Thank you for considering our paper for publication in PlosOne.

You will find below our responses to each point raised by the academic editor and reviewer(s).

Best regards

# Academic Editor’s comments

1. Please ensure that your manuscript meets PLOS ONE's style requirements, including those for file naming. The PLOS ONE style templates can be found at

<https://journals.plos.org/plosone/s/file?id=wjVg/PLOSOne_formatting_sample_main_body.pdf> and

<https://journals.plos.org/plosone/s/file?id=ba62/PLOSOne_formatting_sample_title_authors_affiliations.pdf>

*We have done so*

2. Thank you for submitting your clinical trial to PLOS ONE and for providing the name of the registry and the registration number. The information in the registry entry suggests that your trial was registered after patient recruitment began. PLOS ONE strongly encourages authors to register all trials before recruiting the first participant in a study.

As per the journal’s editorial policy, please include in the Methods section of your paper:

1) your reasons for your delay in registering this study (after enrolment of participants started)

*All the protocols were originally written in French. The submission to clinicaltrials.gov needed translation of our protocol and the review process went late because of administrative delay in our hospital and unavailability of some actors participating in the consensus of decisions regarding the protocol. We decided not to delay the research, and the manuscript makes it plain that study approval by the ethics committee was in March of 2018 and procedures began in August of 2018. Registration at "clinicaltrials.gov" is not mandated by Tunisian law, which covered all legal aspects of this study.*

2) confirmation that all related trials are registered by stating: “The authors confirm that all ongoing and related trials for this drug/intervention are registered”.

Please also ensure you report the date at which the ethics committee approved the study as well as the complete date range for patient recruitment and follow-up in the Methods section of your manuscript.

*We have done so*

3. Thank you for including your ethics statement: "ethics committee mongi slim university hospital , la marsa tunisia

approval number 05/2018

written consent"

Please amend your current ethics statement to confirm that your named institutional review board or ethics committee specifically approved this study.

*We have done so*

Once you have amended this/these statement(s) in the Methods section of the manuscript, please add the same text to the “Ethics Statement” field of the submission form (via “Edit Submission”).

For additional information about PLOS ONE ethical requirements for human subjects research, please refer to <http://journals.plos.org/plosone/s/submission-guidelines#loc-human-subjects-research>.

4. Thank you for stating the following in the Competing Interests section:

"NO authors have competing interests"

We note that one or more of the authors are employed by a commercial company: Ramsay Healthcare France.

*The Ramsay Healthcare Group is a private hospital group. It is not a simple commercial company, but a company owning several private hospitals in Europe. The doctors who work in these hospitals are principally not salaried employees but liberal doctors, and they are not necessarily financed by the private hospital for their research work. In this case, none of the doctors received any funding from Ramsay Healthcare.*

4.1. Please provide an amended Funding Statement declaring this commercial affiliation, as well as a statement regarding the Role of Funders in your study. If the funding organization did not play a role in the study design, data collection and analysis, decision to publish, or preparation of the manuscript and only provided financial support in the form of authors' salaries and/or research materials, please review your statements relating to the author contributions, and ensure you have specifically and accurately indicated the role(s) that these authors had in your study. You can update author roles in the Author Contributions section of the online submission form.

Please also include the following statement within your amended Funding Statement.

“The funder provided support in the form of salaries for authors [insert relevant initials], but did not have any additional role in the study design, data collection and analysis, decision to publish, or preparation of the manuscript. The specific roles of these authors are articulated in the ‘author contributions’ section.”

If your commercial affiliation did play a role in your study, please state and explain this role within your updated Funding Statement.

*None of the authors are salaried of Ramsay Healthcare, but doctors affiliated with Ramsay Healthcare hospitals work as liberal doctors and did not receive any funding for this research protocol.*

4.2. Please also provide an updated Competing Interests Statement declaring this commercial affiliation along with any other relevant declarations relating to employment, consultancy, patents, products in development, or marketed products, etc.

Within your Competing Interests Statement, please confirm that this commercial affiliation does not alter your adherence to all PLOS ONE policies on sharing data and materials by including the following statement: "This does not alter our adherence to PLOS ONE policies on sharing data and materials.” (as detailed online in our guide for authors http://journals.plos.org/plosone/s/competing-interests) . If this adherence statement is not accurate and there are restrictions on sharing of data and/or materials, please state these. Please note that we cannot proceed with consideration of your article until this information has been declared.

*None of the authors are salaried of Ramsay Healthcare, but doctors affiliated with Ramsay Healthcare hospitals work as liberal doctors and did not receive any funding for this research protocol nor have any competing interest. This does not alter our adherence to PLOS ONE policies on sharing data and materials.*

Please include both an updated Funding Statement and Competing Interests Statement in your cover letter. We will change the online submission form on your behalf.

Please know it is PLOS ONE policy for corresponding authors to declare, on behalf of all authors, all potential competing interests for the purposes of transparency. PLOS defines a competing interest as anything that interferes with, or could reasonably be perceived as interfering with, the full and objective presentation, peer review, editorial decision-making, or publication of research or non-research articles submitted to one of the journals. Competing interests can be financial or non-financial, professional, or personal. Competing interests can arise in relationship to an organization or another person. Please follow this link to our website for more details on competing interests: http://journals.plos.org/plosone/s/competing-interests

*We included both an updated Funding Statement and Competing Interests Statement in our cover letter, thank you for changing the online submission form on our behalf.*

5. We note that you have indicated that data from this study are available upon request. PLOS only allows data to be available upon request if there are legal or ethical restrictions on sharing data publicly. For information on unacceptable data access restrictions, please see <http://journals.plos.org/plosone/s/data-availability#loc-unacceptable-data-access-restrictions>.

In your revised cover letter, please address the following prompts:

a) If there are ethical or legal restrictions on sharing a de-identified data set, please explain them in detail (e.g., data contain potentially identifying or sensitive patient information) and who has imposed them (e.g., an ethics committee). Please also provide contact information for a data access committee, ethics committee, or other institutional body to which data requests may be sent.

b) If there are no restrictions, please upload the minimal anonymized data set necessary to replicate your study findings as either Supporting Information files or to a stable, public repository and provide us with the relevant URLs, DOIs, or accession numbers. Please see <http://www.bmj.com/content/340/bmj.c181.long> for guidelines on how to de-identify and prepare clinical data for publication. For a list of acceptable repositories, please see <http://journals.plos.org/plosone/s/data-availability#loc-recommended-repositories>.

*Upon review of these statements, we agree that our data has been sufficiently anonymized and provide it to plosone for unrestricted public access*

We will update your Data Availability statement on your behalf to reflect the information you provide.

6. Your ethics statement should only appear in the Methods section of your manuscript. If your ethics statement is written in any section besides the Methods, please delete it from any other section.

*We have done so.*

7. Please include captions for your Supporting Information files at the end of your manuscript, and update any in-text citations to match accordingly. Please see our Supporting Information guidelines for more information: http://journals.plos.org/plosone/s/supporting-information.

*We have done so.*

# Reviewers' comments:

Reviewer's Responses to Questions

**Comments to the Author**

1. Is the manuscript technically sound, and do the data support the conclusions?

The manuscript must describe a technically sound piece of scientific research with data that supports the conclusions. Experiments must have been conducted rigorously, with appropriate controls, replication, and sample sizes. The conclusions must be drawn appropriately based on the data presented.

Reviewer #1: No

Reviewer #2: Yes

2. Has the statistical analysis been performed appropriately and rigorously?

Reviewer #1: Yes

Reviewer #2: Yes

3. Have the authors made all data underlying the findings in their manuscript fully available?

The [PLOS Data policy](http://www.plosone.org/static/policies.action#sharing) requires authors to make all data underlying the findings described in their manuscript fully available without restriction, with rare exception (please refer to the Data Availability Statement in the manuscript PDF file). The data should be provided as part of the manuscript or its supporting information, or deposited to a public repository. For example, in addition to summary statistics, the data points behind means, medians and variance measures should be available. If there are restrictions on publicly sharing data—e.g. participant privacy or use of data from a third party—those must be specified.

Reviewer #1: No

Reviewer #2: Yes

4. Is the manuscript presented in an intelligible fashion and written in standard English?

PLOS ONE does not copyedit accepted manuscripts, so the language in submitted articles must be clear, correct, and unambiguous. Any typographical or grammatical errors should be corrected at revision, so please note any specific errors here.

Reviewer #1: Yes

Reviewer #2: Yes

5. Review Comments to the Author

Please use the space provided to explain your answers to the questions above. You may also include additional comments for the author, including concerns about dual publication, research ethics, or publication ethics. (Please upload your review as an attachment if it exceeds 20,000 characters)

Reviewer #1: The authors present the results of a single center randomized trial of two different cesarean delivery techniques to determine if the French AmbUlatory cesarean (FAUCS) technique resulted in significantly lower postoperative mean pain scores than a more standard technique used at their hospital, the Misgav Ladach (MLCS) approach. Other outcomes included a combined pain and medication score, total surgical duration, calculated blood loss, maternal autonomy which included measures of time to urination, standing and eating meals, and length of stay. One of two surgeons performed all procedures. Unfortunately, the anesthetic techniques differed between the two groups. The authors conclude that the FAUCS technique “can reduce postoperative pain and accelerate recovery” when compared to the MLCS approach.

Questions and comments for the authors.

1. Inclusion/Exclusion criteria. The general summary statement on the [Clinicaltrials.gov](http://clinicaltrials.gov) site states that women at 36 weeks’ gestation and greater were eligible. On the [Clinicaltrials.gov](http://clinicaltrials.gov) site there is also a link to PDF titled, “Study Protocol and Statistical Analysis Plan”. In this document, the inclusion criteria section states that women 37 weeks’ and greater were eligible. Next, the manuscript does not specifically state the gestational age at which women were eligible. Please add to the manuscript the gestational age inclusion criteria and clarify the discrepancy between the summary on the [Clinicaltrials.gov](http://clinicaltrials.gov) site and that in above-referenced study protocol PDF, which is a link on the [CT.gov](http://ct.gov) site. The manuscript also does not list the age that women had to be in order to be eligible ([CT.gov](http://ct.gov) site states 18-48 years).

*In this study, only women 37 weeks’ and greater were enrolled. We corrected this information on the general summary statement on the Clinicaltrials.gov site.*

*We detailed the required inclusion criterias in the methods section (statements about women age and gestational age are added).*

2. Randomization scheme. The Materials and Methods section of the manuscript (page 5) states that “The two comparison groups were generated using simple randomization, with an equal allocation ratio, by referring to Kendall and Smith’s Table of Random Sampling Numbers [11]”. I went to the referenced web site and the text book was not available on the listed link. Please provide more explanation on the randomization scheme. What were the block sizes? Were blocks used or was the randomization just alternating group assignments. This is important because if blocks were not used and the randomization was just simple alternating numbers, the providers could guess which technique was next and it is possible then that the subject could have been informed of the allocation group.

*We now address this.*

*Two blocks of 50 were used to generate randomization, using the Kendall and Smith published methods. The randomization was NOT just simple alternating numbers, and the providers could NOT guess which technique was next.*

3. Blinding. The second paragraph of page 5 states that you performed an “unblinded randomized clinical trial” but then on page 6, in the first paragraph you state “participants, residents involved in patient care, and all investigators were blinded”. These two statements seem contradictory. I appreciate that the surgical team cannot be blinded to the cesarean but then you state that “all investigators were blinded”. If the surgeons are co-authors on the manuscript, then I would consider them as investigators. Next, were residents who performed the post-operative care not present or scrubbed for the surgery? Please clarify the statements about blinding. I worry greatly that patients could have discovered which group assignment they were in and if they then also knew that FAUCS was the intervention being tested, that they would then want to report improved pain scores.

*We now clarify this.*

4. Anesthetic plan. Each group received a different anesthetic regimen. You acknowledge this limitation (page 16), but I believe this to be a significant weakness in your study. The anesthetic regimens may be driving your outcomes rather than the surgical procedure. By having different anesthetic regimens between the two groups, you lose the benefit of performing a randomized trial that attempts to test the difference between two surgical techniques. With this design, it is impossible to know if the different surgical techniques are driving the findings, or the different anesthetic techniques. This limitation needs to be highlighted throughout the text, including any concluding statements in the Abstract and Discussion (and title) sections which state that the FACUS resulted in improved postoperative pain.

*We now address anesthesia.*

5. Surgical techniques. Neither the MLCS nor the FAUCS techniques are commonly used in the United States. You provide some references to the techniques and briefly describe them but my preference is that more information be provided on these techniques. Can you work with the Academic Editor on this? Maybe you could include illustrations of the two techniques as Supplemental material. This added information would help obstetricians in the United States better understand the two techniques. Most obstetricians in the US use either a Pfannenstiel (with lateral rectus sheath fascial incisions) or a Joel-Cohen technique.

*The MLCS is also called the Joel-Cohen technique (Joel Cohen described a technique for hysterectomy, and Michael Stark applied it to cesarean section. Both were working in Misgav Ladach hospital).*

*We would be happy to provide supplementary material to illustrate both techniques, but we would need at least 4 weeks more to provide original pictures for it.*

6. One of the secondary outcomes was listed as “combined pain/medical score”. Which analgesic agent was used for the combined pain/medical score calculation? Earlier in the manuscript, you state that your standard postoperative analgesic plan was intra-rectal ketoprofen and then intravenous paracetamol as second line. Did you just use the ketoprofen medication dose in the calculation used for this secondary outcome? Please clarify.

*We used the ketoprofen dose and now clarify this.*

7. The title states that the FAUCS “leads to superior maternal outcomes”. I would clarify this statement and make it more specific; i.e. improved pain scores. Also see my concerns outlined in #4.

*We changed the title for “The extraperitoneal French AmbUlatory cesarean section technique leads to improved pain scores and a faster maternal autonomy compared with the intraperitoneal Misgav Ladach technique: A prospective randomized controlled trial”*

8. You state that your primary outcome is a mean of the five VAS scores performed at postoperative time points, H0, H6, H12, H18 and H24. These five time points were never explicitly defined. I appreciate that they were performed every six hours over the course of 24 hours but when did they start? What is H0? Please clarify.

*H0 designated the end of surgery time . We now clarify this .*

9. Table 2 and Figure 2 provide mean VAS scores at each of the five time points. The y-axis of the figure and the table legend both use the abbreviation “PMPS”. This is technically not correct. You define the PMPS as the average of the five time points while the figure and table provide the mean of each of the individual VAS scores at each time point. Please clarify and adjust the Figure axes and Table legend to clarify that you are presenting mean VAS scores at each time point and not your defined PMPS.

*We now clarify and adjust the figure axes*

10. Please provide p-values in Tables 2 and 3 so the reader can determine if the differences in mean VAS scores (Table 2) and autonomy scores (Table 3) are significantly different at each time point. This is particular important as you claim that the FAUCS results in improved pain and autonomy.

*The reviewer may not have identified correctly the models used. They were not ANOVA that presume each time point was an independent value of a multinomial variable. Instead, time points were treated as what they were, to wit, quantities. This analysis indicated specific and progressive differences* ***over time****, with time as a continuous variable. It would not have been possible to have a polynomial of time in the model were it not treated as a quantity. The reviewer is asking for an analysis that we cannot perform because it is innapropriate. To help the reviewer interpret, the significance of the “Technique” variable indicates that the techniques differed over the entire timeframe, and the interaction of “Technique” and “Time” indicates that the trajectories differ. Likewise, Cox proportional hazards models analyze time-to-event treating time as quantitative units and are not appropriate for timepoint-by-timepoint analysis.*

11. Table 1 lists Test statistic values, p-values, R2 values and raw p-values. These can all be excluded from the table given that your performed a randomized trial. The Academic Editor can clarify if the journal requires these values.

*We would be happy to comply, but we wish to remain cautious and consult with the Academic Editor.*

12. The first full paragraph of the Page 10 (Results section) provides the differences in “postoperative pain” between the two groups. Is this your PMPS score? If so, please clarify.

*Yes, we now clarify in the text.*

13. I am confused about the secondary outcome that is labeled “maternal autonomy”. You attempt to define this outcome in the ‘Study Outcomes’ paragraph on page 7 but the definition is still not clear. You state that maternal autonomy was “calculated as a summation of postoperative times to spontaneous urination, standing, or first meal in hours”. Please provide more information on how this variable was calculated. Was it the amount of time from the conclusion of the surgery to any one of those three outcomes? The inclusion of the word “or” suggests that it was the time to any one of the three but you also use the word ‘summation’, which suggests it was the total time to all three. Was the outcome; autonomy = (time from end of surgery to urination) + (time from end of surgery to unassisted standing) + (time from end of surgery to first meal)? Were foley catheters removed at the same duration of time following surgery for all subjects? If not, the variation in when the catheters were removed could affect this outcome rather than the surgical intervention. The delivery of food trays could also be varied based on what time of day the surgery was performed. Since you don’t feed women until they pass flatus, why did you use eating as the outcome variable rather than passing flatus?

*It was not a summation. The correct description would be whichever came last. We now correct this.*

*We don’t feed women until they pass flatus, but we feed them as soon as they pass flatus. For that reason, the available parameter was the time of food distribution.*

*Foley catheters were placed depending on incapacity to urinate and removed at the first unassisted standing for all surgeries, so we didn’t find this information to impact autonomy evaluation, compared to the time from end of surgery to unassisted standing.*

14. The last sentence on page 11 states, “Instrument assistance was necessary in 84% of FAUCS procedures (p<0.001)”. What instruments are you referring to? Next, what is the p-value comparing this outcome to? Is it for the use of instruments in the MLCS group?

*We corrected the citation in the text :*

*Results*

*Instrument assistance using Wrigleys’ forceps or Teissiers’ spatulas was necessary in 84% of FAUCS procedures (p < 0.001). The hypothesis tested was that FAUCS would result in increased instrument assistance, as FAUCS incisions are smaller and instrument assistance usually described as part of the technique.*

*Discussion*

*We recommend some tips for reducing extraction time while performing FAUCS, such as: (1) opening the anterior rectus sheath with sufficient width up and down; (2) reclining the left rectus abdominis muscle correctly to the left; and (3) involving the mother in the process by having her control her breathing [4,22] during the use of small guiding instruments (e.g., Wrigley forceps or Teissier spatulas) [23] which allows to perform smaller incisions. This instrument assistance is usually very easily and gently performed, while there is no bony obstacle to the exit of the newborn, but only soft tissues around.*

15. The second paragraph of the Discussion section refers to better overall birth experience and lower financial costs. Neither of these outcomes were studied, thus I would suggest that these claims are removed from the manuscript.

*We have done so.*

16. I may have missed it, but I did not see that you reported the 'combined pain/medication score' outcome data that was a planned secondary outcome.

*We addressed the mistake in the text. we reported PAMS scores in the tables and the figures.*

17. The third paragraph of the Discussion includes a statement, “We found that FAUCS was so effective at reducing surgical pain that early rehabilitation depended more on preserved maternal autonomy than on painkillers and protocols”. I do not understand this statement. Next, given that the two groups received different anesthetic protocols, you do not know if the differences in postoperative pain scores that were seen were due to the surgical technique or the anesthetic technique.

*We have cut this “enthusiastic” statement.*

18. CONSORT. Please state that the full protocol is available on the [Clinicaltrials.gov](http://clinicaltrials.gov) site. See my concerns above about randomization and blindings.

*We now do so.*

Reviewer #2: This is the report of a randomized clinical trial of Cesarean techniques (I think Cesarean should be capitalized throughout, incidentally). The primary outcome is a VAS composite score. The authors need to reference their score functions, or at least explain why they are appropriate. The randomization procedure is described, statistical analysis is sophisticated with longitudinal and survival models, and limitations of the study are clearly enumerated. The study statistician uses Holm's method to adjust for multiplicities. From a statistical standpoint, the authors are to be congratulated. There are two glaring omissions that would be useful to correct in a revision:

*We have clarified why VAS seemed appropriate.*

1. Sample size is not really described. 30% of what? What are the assumptions? Is this based on the actual analysis technique in the study, or just a simple t-test? In the discussion, the authors should clearly point out if the sample size assumptions were actually realized in the clinical trial so we can figure out if the study was over- or under- powered.

*We completed with the following statements :*

*Materials and Methods*

*Sample size calculation was based on the PMPS. The effect of FAUCS was considered important if the difference in PMPS between groups was ≥ 30%, using a t-test. To achieve 80% power with an α = 0.05, it was anticipated that 45 patients in each group were required.*

*Discussion*

*The sample size assumptions were realized in this clinical trial, with an initial hypothesis of 30% reduction of PMPS, and an effective 36,17% of PMPS reduction observed.*

2. Lots of great regression models, and not a single word about assumptions of the model, or whether these assumptions were tested or met.

*We hope we have adequately addressed this comment.*

6. PLOS authors have the option to publish the peer review history of their article ([what does this mean?](https://journals.plos.org/plosone/s/editorial-and-peer-review-process#loc-peer-review-history)). If published, this will include your full peer review and any attached files.

If you choose “no”, your identity will remain anonymous but your review may still be made public.

**Do you want your identity to be public for this peer review?** For information about this choice, including consent withdrawal, please see our [Privacy Policy](https://www.plos.org/privacy-policy).

Reviewer #1: No

Reviewer #2: No
